# Influence of natural and anthropogenic drivers on plague risk in Southwest China: A multicenter cross-sectional study

**DOI:** 10.1016/j.onehlt.2025.101142

**Published:** 2025-07-25

**Authors:** Zhe Lou, Huajun Zhao, Chao Su, Ennian Pu, Xiyang Li, Qingxi Shi, Yunqin Shen, Ying Zhao, Zihou Gao, Ruiyun Li

**Affiliations:** a School of Public Health, Nanjing Medical University, 101 Longmian AV., Nanjing, Jiangsu 211166, China; b Yunnan Institute of Endemic Diseases Control and Prevention, No. 5 Wenhua Road, Dali, Yunnan, China; c School of Public Health, Dali University, No. 2 Hongsheng Road, Dali, Yunnan 671003, China; d Jiangsu Center for Collaborative Innovation in Geographical Information Resource Development and Application, Nanjing, China

**Keywords:** Plague, Epidemiology, Anthropogenic factors, Living conditions, Ethnic disparities

## Abstract

The natural and anthropogenic environment have contributed to the dynamic risk of plague and their threats to human health. Although evidence has indicated the environmental suitability for disease dynamics, the alteration of the risk by anthropogenic factors have not been fully investigated. We conducted a multicenter cross-sectional survey among 2998 residences across 54 villages in 13 counties in Southwest China. With the survey data, we developed composite anthropogenic indices to make systematic assessment of people's living environment, household sanitation levels, and risk perception regarding plague. We identified disparities of these anthropogenic indices among ethnic groups. By featuring plague, we further established statistical model to assess how environmental and anthropogenic factors associate with the occurrence of the typical zoonotic disease. Our results show that the improved living environment is significantly linked to the lower risk of plague occurrence. In contrast, we have no evidence for the significant association between household sanitation, protective behaviors, environmental conditions and plague risk. These findings pointed at the improved living environment as the most likely anthropogenic driver that is associated with the reduced risk of plague. Integrating anthropogenic modulators of disease dynamics in public health strategies would be the key for the effective management of disease risks.

## Highlights

• We developed composite indices and assess anthropogenic impacts on zoonotic risks.
• Improved living conditions is critical for the management of zoonotic disease risks.
• Ethnic disparities highlight the need for the culturally tailored interventions.

## 1 Introduction

Zoonotic diseases, often originating from wildlife reservoirs, are increasingly threatening human health globally. It has been recognized that natural and anthropogenic factors may have collectively determined the risk of disease circulation [[1], [2], [3], [4], [5], [6]]. Therefore, comprehensive assessments of how the interconnected factors associate with disease risks help to characterize the natural and anthropogenic contexts underlying disease dynamics among local settings.

Noted that the natural and anthropogenic factors were inevitably associated with zoonotic disease dynamics in several ways. Pilot studies have demonstrated that temperature and precipitation regulate the survival and behavior of host animals and vectors, contributing to disease epidemics [3,7,8]. Additionally, soil characteristics influence ecological characteristics of vectors and may have the potential to be the reservoir of pathogens [[9], [10], [11], [12]]. Apart from ecological conditions, anthropogenic drivers such as socioeconomic development and human behaviors have also altered disease transmission. Socioeconomic disparities critically influence the dynamics of disease transmission by affecting healthcare accessibility, living environment, and sanitation conditions, thereby generating uneven vulnerability across populations [[13], [14], [15], [16]]. Human activities modified wildlife-human interactions and proximity to contaminated environments, increasing the likelihood of zoonotic spillovers [17,18]. Indeed, existing evidence has indicated that the living space of humans and hosts has been increasingly overlapping [19,20].

In the rapid process of urbanization [20,21], evaluating people's perception of zoonotic disease risks and knowledge of disease prevention and control would be fundamental to improving the effective management of the disease. Studies have shown that socioeconomic and behavioral factors strongly influence zoonotic disease risk. In Zambia, traditional practices such as rodent hunting have been linked to increased plague exposure [22]. In China, HFRS incidence was associated with education and population density [23]. Historical evidence further indicates that lower socioeconomic status consistently correlates with higher infectious disease mortality [24], highlighting the importance of integrating social context into zoonotic disease prevention. However, existing evidence have been biased towards the influence of ecological and environmental factors on emerging and re-emerging zoonotic diseases. The lack of quantitative assessments regarding current living environment, household sanitation levels, risk perception, and attitude towards protective measures limits our effort to examine the link between the changing anthropogenic factors and plague circulation risk.

Plague is a natural epidemic disease caused by *Yersinia pestis* and is primarily transmitted to humans through the bite of infected fleas and direct contact with infected animals and individuals [25,26]. Although the disease has been effectively controlled, rodent plague epizootics remain active and cyclic due to ecological stability [27]. It re-emerged among local settings in Yunnan Province, southwest China, where plague has historically played a major role, the origin of the third pandemic in the late 19th century. From 1950 to 2020, Yunnan reported 3464 human plague cases with a fatality rate of 18.4 %, and over 4900 *Y. pestis* isolates from hosts and vectors, reflecting sustained animal-to-human transmission risk [28,29]. The abundant natural resources and the close animal-human interaction in the typical epidemic foci have presented the ongoing challenges to disease management. Therefore, developing effective strategies to mitigate the risk of future outbreaks requires the critical investigation of how natural and anthropogenic conditions influence disease dynamics. However, large-scale investigations into the influence of anthropogenic factors on plague dynamics are limited. Such that the knowledge gap regarding the associations between human activities and plague occurrence poses significant challenges to the design of targeted preventive strategies.

In this study, we conduct a large-scale and multicenter survey across 54 villages located in 13 counties in Southwest China. We explicitly address the comprehensive assessment of the living environment, household sanitation levels, and risk perception of people by proposing composite anthropogenic indices. We further identify the disparities of these anthropogenic factors among villages' urbanicity and ethnic groups. Additionally, we examine their association between anthropogenic environment with the risk of plague occurrence.

## 2 Methods

### 2.1 Study design

This multicenter cross-sectional survey was conducted across 54 villages in the Southwest China between September 15, 2023 and January 21, 2025. These villages located among 13 counties in the Yunnan Province (i.e. Lianghe, Yulong, Yongde, Menglian, Mengla, Menghai, Jianshui, Mojiang, Gengma, Yuanyang, Yiliang, Yanshan and Maguan), covering a large variety of environmental and socioeconomic conditions. Respondents were recruited primarily by calling upon villagers by village doctors and postings on the village notice board.

### 2.2 Population survey data

The survey was conducted using a questionnaire carefully formulated by experts following thorough discussions and researches. The questionnaire was consisted of 36 across five parts, featuring a mix of binary, multiple-choice, and frequency-scale questions to investigate demographic information of respondents, plague epidemic situation, living environment, plague prevention awareness, and physical health condition. The survey data was subsequently collected by a program developed by the research team. The questionnaire was translated to English version and provided in the supplementary material.

### 2.3 Meteorological and environmental data

We acquired daily climate data, including 2-m air temperature, total precipitation, leaf area index for high vegetation, leaf area index for low vegetation, and the layered soil temperature and volume of water, from the fifth-generation reanalysis (ERA5) provided by the European Center for Medium-Range Weather Forecasts (ECMWF) [30]. Time-series data of the meteorological and environmental factors were extracted by identifying the nearest grid point to the center of each local authority. This data was then aggregated to yield the yearly average temperature, leaf area index for low vegetation, and yearly total precipitation for each village. To further evaluate the climate conditions of each village in recent years, these data were summarized into a 5-year average from 2020 to 2024. The 5-year average was applied because: (1) plague endemicity was assessed at the village level as a binary variable based on multi-year surveillance to account for the low frequency and irregular timing of outbreaks. (2) the socioeconomic and anthropogenic factors of primary interest in our study typically exhibit stability over these timescales; and (3) longer-term averages help buffer against interannual variability that might obscure persistent environmental patterns relevant to plague maintenance.

### 2.4 Composite indices

The anthropogenic environment was characterized in terms of the overall living environment, household sanitation levels, and protective behavior. Aligned with this, we built three composite indices by integrating responses to the related survey questions (Table S1). For each index, questions involved were initially standardized by using eq. (1):

(1) $$d_{\mathrm{ij},\mathrm{pos}}=\frac{x_{\mathrm{ij}}-x_{\mathrm{ij},\min}}{x_{\mathrm{ij},\max}-x_{\mathrm{ij},\min}}\;\text{or}\;d_{\mathrm{ij},\mathrm{neg}}=\frac{x_{\mathrm{ij},\max}-x_{\mathrm{ij}}}{x_{\mathrm{ij},\max}-x_{\mathrm{ij},\min}}$$

where $d_{\mathrm{ij}}$ was the standardized value of variable $j\left(j=1,\ldots ,4\right)$ for index $i\left(i=1,\ldots ,3\right)$. Consistently, $d_{\mathrm{ij},\mathrm{pos}}$ and $d_{\mathrm{ij},\mathrm{neg}}$ represent the standardized values for positive and negative responses, respectively. $x_{\mathrm{ij}}$ represented the original, non-standardized value of variable j for index i; and accordingly, $x_{\mathrm{ij},\max}$ and $x_{\mathrm{ij},\min}$ was the maximum and minimum value of the original data for variable j, respectively

Next, we derived the weights of each variable by evaluating the entropy of the standardized responses of each question using the Entropy Weighting Method (EWM) [31] (eqs. (2), (3), (4)). EWM characterizes the relative variability and informativeness of the responses. Therefore, the weight was minimized when responses were similar among individuals and increased as it focused on particular responses.

(2) $$P_{\mathrm{ij}}=\frac{d_{\mathrm{ij}}}{\sum_{j=1}^{n}d_{\mathrm{ij}}}$$

(3) $$e_{\mathrm{ij}}=-\frac{1}{\ln n}\sum_{j=1}^{n}P_{\mathrm{ij}}\ln P_{\mathrm{ij}}$$

(4) $$W_{\mathrm{ij}}=\frac{1-e_{\mathrm{ij}}}{\sum_{j=1}^{n}1-e_{\mathrm{ij}}}$$

where $P_{\mathrm{ij}}$ is the fraction of standardized value of variable j among the standardized values of all the n variables for index i. $P_{\mathrm{ij}}$ is further translated to quantify the entropy ($e_{\mathrm{ij}}$) of variable j and the corresponding weight ($W_{\mathrm{ij}}$) using EWM.

The composite index was calculated as a weighted sum of the standardized responses using $U_{i}=\sum_{j=1}^{n}W_{\mathrm{ij}}\times d_{\mathrm{ij}}$. $U_{i}$ is the comprehensive value of dimension i which is determined by $d_{\mathrm{ij}}$ and $W_{\mathrm{ij}}$ accordingly. These composite indices allowed us to make quantitative assessment of the anthropogenic environment, indentifying the dispatrities of the living environment, hygiene standards, and protective behavior across population subgroups.

### 2.5 Statistical analyses

We excluded 13 respondents with missing, incompleted or repeated personal information such as residential address. All subsequent analyses were conducted based on the remaining 2998 valid questionnaires. Recognizing the diverse demographic characteristics, we distinguished the villages by the major ethnic groups (categorized as Han or ethnic minority). To facilitate the subsequent statistical analyses, we also distinguished the urban and rural areas of the village, in accordance with the latest official urban-rural code table from the National Bureau of Statistics (https://www.stats.gov.cn/). Plague endemic status of each village was determined based on surveillance data provided by the Yunnan Institute of Endemic Diseases Control and Prevention.

We started with descriptive analyses of demographic characteristics of the survey population. Chi-square test and Kruskal-Wallis non-parametric test were then used to test the differences in baseline population indicators between ethnic groups. For the three composite indices, independent samples *t*-tests and Wilcox rank-sum tests were applied to compare differences between regions that whether experienced plague outbreaks, as well as between urban and rural areas. One-way analysis of variance (ANOVA) and the Kruskal-Wallis non-parametric test were employed to compare differences among ethnic groups. Furthermore, to assess the association between those factors and the plague endemic status of the village in recent years, we fitted generalized linear mixed models (GLMM) with a binomial distribution, using the plague endemic status of villages as response variable (endemic/non-endemic). Living environment, household sanitation conditions, and protective behavior as explanatory variable. Yearly average temperature and number of precipitation days for each village as covariates. We included the ethnic as a random factor to account for the spatial and temporal variability.

Statistical analysis was conducted using Microsoft Excel 2019 and R Statistical Software (v4.3.3; R Core Team) in RStudio. All statistical tests were two-tailed and statistical significance was assumed for a *p* < 0.05.

## 3 Results

### 3.1 Study population

**Fig. 1:**
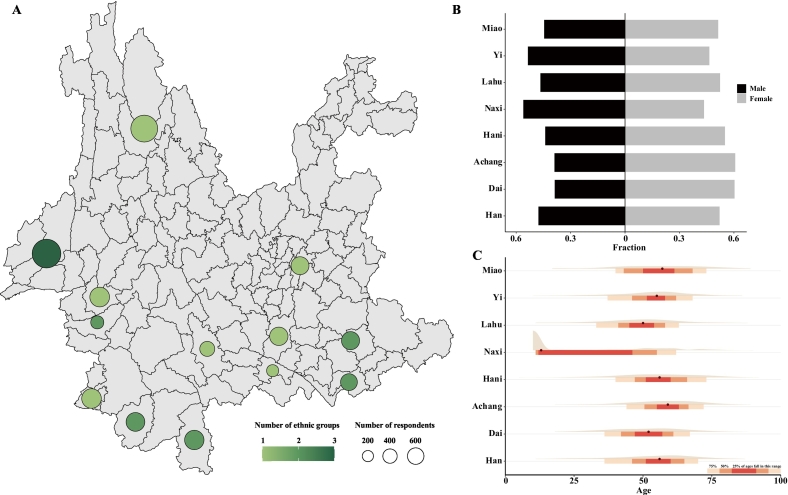
Disparities of the sociodemographic characteristics across ethnic groups. (A) County-level number of respondents and ethnic groups involved in the survey. Circle size and color indicates the number of respondents and ethnic groups in each county, respectively. The (B) fraction of male and female respondents and (C) median age are stratified by ethnic groups.

A total of 2998 respondents from eight ethnic groups residing in 54 villages among 13 counties were interviewed over the course of the study, averaging approximately 56 individuals per village (Fig. 1A). Although the fraction of individuals varied by counties, we involved an equivalent number of male (46.96 %, *n* = 1408) and female (52.74 %, *n* = 1573) respondents. The median age of these respondents was 53 years old, whereas over half of the respondents were aged 40–69 years old. The respondents were mainly farmers (2319 of 2998, 77.35 %), followed by students (312, 10.14 %), and others were businessmen, workers, medical personnel, service workers, teachers, and retirees. Moreover, around 80 % of all the respondents (2334 out of 2998) raised animals, and in particular 24.29 % (567 of 2334) raised animals at home. The details of sociodemographic characteristics of the respondents are provided in Table 1.

**Table 1:** Sociodemographic characteristics of the study respondents (*n* = 2998).

Characteristics	n (%)
Gender	
Woman	1573 (52.47 %)
Man	1408 (46.96 %)
Missing data	17 (0.57 %)
Age, years	
≤19	308 (10.27 %)
20–29	125 (4.17 %)
30–39	245 (8.17 %)
40–49	520 (17.34 %)
50–59	774 (25.82 %)
60–69	626 (20.88 %)
70–79	290 (9.67 %)
≥80	60 (2.00 %)
Missing data	50 (1.68 %)
Occupation	
Farmer	2319 (77.35 %)
Student	312 (10.41 %)
Others	338 (11.27 %)
Missing data	29 (0.97 %)

It is noted that sociodemographic characteristics varies across ethnic groups (Table S2). Of the eight ethnic groups involved in our study, Naxi group exhibited a higher proportion of males as compared to other groups, with significant differences (adjusted *p* < 0.05) found between the Naxi and Dai, Hani, and Achang groups. Similarly, the Yi group also showed a significantly higher proportion of males as compared to the Dai and Achang groups (adjusted *p* < 0.05) (Fig. 1B, Table S3). Additionally, we identified significant disparities of the age profile among ethnic groups (Fig. 1C, Table S4, Supplementary Results). Naxi and Lahu respondents exhibited significantly younger age profiles (adjusted *p* < 0.05). The median age of Naxi group is 13 which is lower than other groups. Similar demographic characteristics is also observed among 315 respondents of the Lahu group, with a median age of 50 which is younger than the Achang, Hani, Yi, Han, and Miao groups (adjusted *p* < 0.05). Moreover, there were significant variations in animal-raising practices among respondents from different ethnic groups (*p* < 0.05) (Fig. S1, Table S5). Our survey shows that animal-raising practices is more prevalent among respondents from Naxi and Hani group as compared with those form the Dai, Yi, Lahu, Han, and Achang groups (adjusted *p* < 0.05). In contrast, respondents from the Miao group are associated with a similar proportion of raising animals compare to those from the other ethnic groups. These findings allow us to involve disparities of animal-raising practices among ethnic groups in further investigation of the anthropogenic factors related to plague epidemiology.

### 3.2 Risk perception and preventive practices of plague infection

Direct exposure to the primary risk factors of plague among respondents was relatively low but not negligible. Our results showed that around 10 % (*n* = 319) of the respondents reported exposure history with found dead rodents. Consistently, 15.34 % (*n* = 460) of the respondents confirmed experiencing flea bites. We also showed that there were approximately 0.60 % (*n* = 18) individuals who had contact with a person who had already contracted plague (Table S6).

**Fig. 2:**
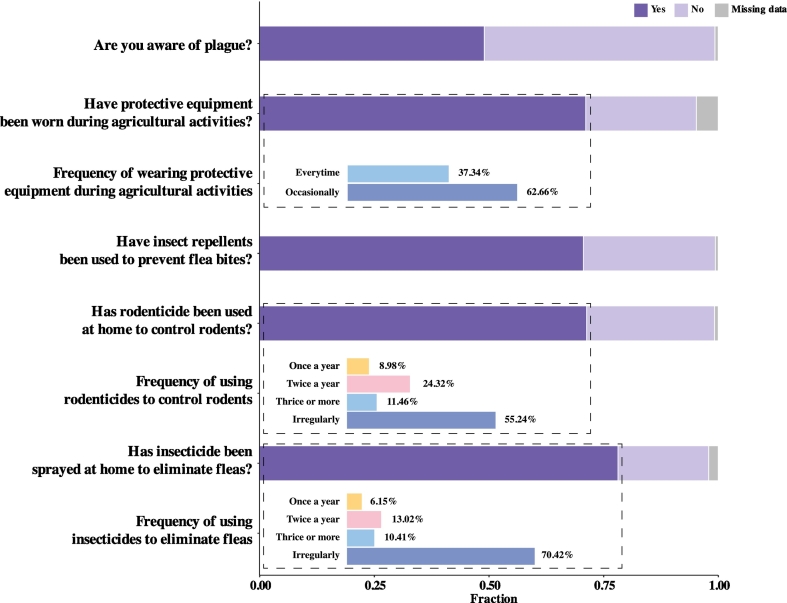
Protective behaviors and risk perception of plague among respondents. Each panel represents the fraction of respondents reporting specific behaviors or practices. Stacked bars represent binary responses for questions regarding protective behaviors and risk perception of plague. For the respondents who answered “Yes”, details of the frequency of the behaviors or practices are provided by grouped bars.

Critically, our assessment of people's risk perception identifies that around half of the respondents (49.00 %, 1469 out of 2998) are aware of plague. This indicates a significant portion of the population with insufficient risk perception of the disease. Consistent with risk perception, our results showed implementation of several preventive practices against plague infection among most respondents. More specific, 70.61 % (*n* = 2117) of respondents reported using insecticides or repellents to prevent infections from flea bites during agricultural activities. Personal protective equipment such as rain boots during agricultural activities were commonly or occasionally used among the two-thirds (71.11 %, *n* = 2132) of the respondents, albeit 24.42 % (*n* = 723) did not take any protective measures. It is also noted that household preventive activity against fleas and rodent were commonly used among respondents. Our investigation shows that spraying insecticides against fleas at home were carried out among 80 % (*n* = 2343) of respondents with varying frequencies. There was around one-third of the respondents reported regular prevention of fleas at home, with 6.15 % (*n* = 144) of them performed the activity once a year, while 13.02 % (*n* = 305) twice a year, and 10.41 % (*n* = 244) three or more times. Comparatively, the majority of respondents with household preventive activities (70.42 %, *n* = 1650) reported spraying insecticides against fleas at home are when fleas were present. For rodent control, over 70 % (*n* = 2138) of respondents had used rodenticides in their houses, with 8.98 % (*n* = 192) using them once a year, 24.32 % (*n* = 520) twice a year, 11.46 % (*n* = 245) three or more times annually, and 55.24 % (*n* = 1181) using them irregularly (Fig. 2).

### 3.3 Disparities of anthropogenic environment

**Fig. 3:**
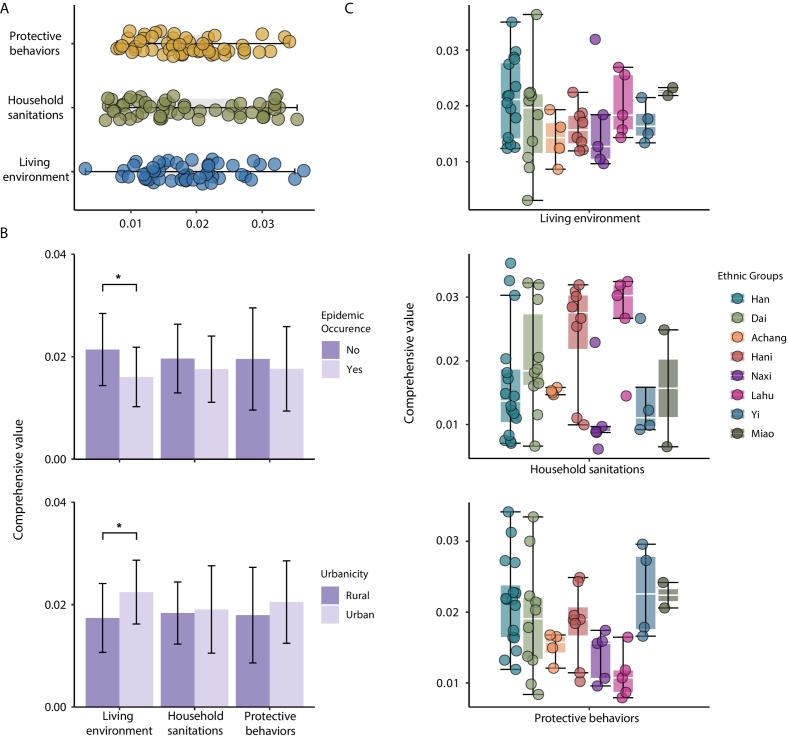
Comprehensive anthropogenic indices of living environment, household sanitation, and protective behavior. (A) Estimates of anthropogenic indices across villages. (B) Disparities of the indices between villages with and without endemic plague circulation, as well as the urban and rural settings. (C) Disparities of the indices across ethnic groups. Statistically significant results (*p* < 0.05) are marked with *.

The composite anthropogenic indices well characterized the living environment, household sanitation and protective behavior among villages, with median estimates of 0.018 (IQR, 0.013–0.022), 0.016 (IQR, 0.011–0.027), and 0.018 (IQR, 0.013–0.022), respectively (Fig. 3A). In view of the weights of the indices, our results demonstrated that house structure predominates the level of living environment, while the ownership of livestock and use of rodenticides largely determined the status of household sanitation and people's protective behavior, respectively.

It is noted that there was significant difference of living environment between villages with endemic and non-endemic circulation of plague. Our findings showed a significantly better living environment among respondents living in non-endemic villages as compared with those with endemic plague circulation (*p* < 0.05). Additionally, we observed a significant difference regarding living environment between urban and rural areas (*p* < 0.05), with a higher level of living environment in urban areas (Fig. 3B). In contrast, we detected no evidence of the significant difference in household sanitation and protective behavior along the urban-rural gradient and across settings with varying levels of urbanicity and magnitude of plague circulation.

Importantly, we point at the critical difference in protective behavior among ethnic groups (Table S7). The Lahu ethnic groups was of the significantly lower awareness of protective behavior compare to the Han group (adjusted *p* < 0.05) (Table S8). However, living environment level showed no significant differences among ethnic groups (all adjusted *p* > 0.05), indicating relatively consistent levels across groups in these areas. This pattern underscores protective behavior as the primary area where ethnic group differences emerge, while living environment and sanitation levels appear more homogeneous across groups, in contrast to the significant living environment disparities observed between urban-rural and endemic-non-endemic settings.

### 3.4 Risk factors assessment

**Fig. 4:**
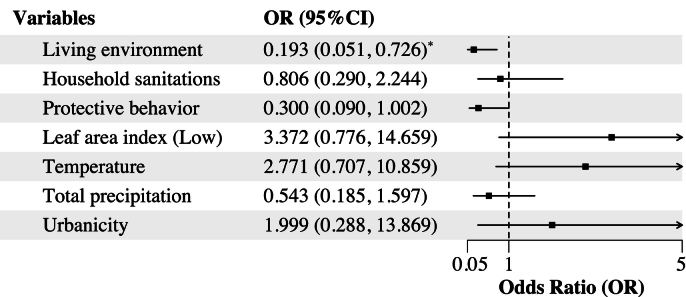
Estimates of the effect of comprehensive indices and environmental factors on the occurrence of plague. Odds ratios (OR) and 95 % confidence intervals (CI) for variables are presented. Significant associations (*p* < 0.05) are marked with *.

The model inference revealed statistical associations between the likelihood of plague occurrence and multifaceted factors. Of these factors, living environment was exclusively associated with plague risk (Fig. 4). For every unit increase of living environment, it is estimated that the risk of plague occurrence decreased by 80.68 % (OR = 0.193, 95 %CI: 0.051, 0.726, *p* < 0.05). It is possible that other natural and anthropogenic factors were associated with plague risk, albeit the association was weak or not statistically significant. Specifically, household sanitation (OR = 0.806, 95 %CI: 0.290, 2.244) and protective behavior measures (OR = 0.300, 95 %CI: 0.090, 1.002) were linked to a reduced risk of plague. Similarly, natural environment such as low vegetation cover in rodent habitat areas (OR = 3.372, 95 %CI: 0.776, 14.659) showed non-significant associations with plague risk. Although ambient air temperature and total precipitation are known to influence vector dynamics, we identified weak association between temperature (OR = 2.771, 95 %CI: 0.707, 10.859) and total precipitation (OR = 0.543, 95 %CI: 0.185, 1.597) with local plague risk.

Disaggregation of the levels of villages' urbanicity and ethnic groups indicated exclusive but weak increase of plague risk in urban areas (OR = 2.00, 95 %CI: 0.29, 13.87). This pattern is possibly associated with the higher population densities and varied sanitation standards and thus the complex and context-dependent influences on plague risk in urban areas. Moreover, the model's random effect for ethnic groups had a variance of 0.883 and a standard deviation of 0.940, suggesting modest variation of plague risk across different ethnic groups. This variation may be attributable to the large variety of cultural practices, accessibility to healthcare facilities, or environmental exposures among ethnic groups.

## 4 Discussion

Our study provides new insights into how natural and anthropogenic factors shape the typical zoonotic disease risk in Yunnan. Importantly, our findings underscore that living environment predominates the plague risk, recognizing disparities of the anthropogenic conditions among ethnic groups.

Our findings highlight that improved living environment play a central role in reducing plague risk, with a 80.68 % reduction in plague likelihood per unit increase in living environment quality. This finding indicates that the enhanced living environment, such as the improved housing quality, may have made substantial contribution to lowering plague risk by facilitating the access to resources and reducing overcrowding among household members. This aligns with prior research linking structural improvements with lower transmission of vector-borne diseases [32,33]. The results point to the importance of addressing anthropogenic disparities as a preventive strategy for plague control. Enhancing living standards in rural areas, particularly in endemic regions, could mitigate exposure to rodent and flea populations, which are key vectors for plague. This outcome emphasizes the need for policies that address structural factors, such as housing quality, resource access, and sanitation infrastructure. By improving these conditions, communities may experience a broader public health impact that extends beyond plague prevention to other infectious diseases linked to similar vectors [34]. In practice, housing improvement efforts in rural Yunnan could prioritize basic structural reinforcement, rodent-proofing, and improved flooring or wall sealing to reduce indoor rodent activity and enhance plague prevention.

Our results regarding household sanitation and protective measures revealed a significant gap between awareness and consistent preventive practices, offering insights into disease prevention and control. Although most villagers were aware of the prevention of rodents and fleas, preventive measures have been carried out irregularly. We identified that over two-thirds of the respondents reported using insecticides or repellents during agricultural activities and rodenticides in their houses. However, these measures were often applied only when rodent or flea populations became noticeable. This irregular application of preventive measures highlights the current challenges to the response of public health emergencies among villages such as the limited access to preventive resources. Public health strategies to improve the regular and proactive control of plague is one crucial direction for future investigations.

Disparities of the plague risk along the urban-rural gradients indicates a slight increase, though non-significant, of the risk in urban areas. This increased risk may be partially related to the higher population density and greater variety of sanitation standards among urban settings [35,36]. Ethnic differences were observed primarily in protective practices rather than living environment, with certain groups displaying distinct behaviors that may influence exposure to plague vectors. A recent study conducted in rural western China during the COVID-19 pandemic also identified a significant behavioral gap between Han and minority ethnic groups, with lower protective behavior scores among minorities largely explained by disparities in household assets, health knowledge, and self-efficacy [37]. These factors may also apply to plague prevention, as limited access to health information, lower health literacy, and reduced confidence in taking preventive action could reduce protective behavior among minority groups. This highlights the importance of tailored interventions that take into account cultural factors, socioeconomic conditions, and local practices. By addressing these nuanced differences, public health strategies can be better adapted to specific community needs, whether in urban or rural settings.

It is noted that we have no evidence on the associations between natural environment on plague risks, although temperature and precipitation are known to influence vector populations. This finding may be largely attributable to the complex and context-dependent influence of environmental factors on plague transmission [[38], [39], [40]]. The impact of climate on plague dynamics may be interrelated and modulated by other factors, such as human socioeconomic behaviors and the unique ecological settings. Additionally, we note that several environmental variables (e.g., leaf area index for low vegetation, precipitation) exhibited wide confidence intervals, suggesting substantial uncertainty rather than definitive effects. These broad intervals may reflect limited statistical power or genuine ecological heterogeneity in how environmental factors influence rodent populations across different microhabitats. These findings suggest that larger-scale environmental triggers, such as seasonal fluctuations or extreme weather events, might play a role that our cross-sectional design was unable to capture. Further research could explore these environmental variables through longitudinal data with higher spatial resolution and temporally resolved exposures, potentially identifying climatic patterns that coincide with peak plague transmission periods. This approach would allow for a more detailed understanding of how environmental factors, in conjunction with socioeconomic conditions, might predict periods of heightened plague risk.

While our findings reveal significant associations, the cross-sectional nature prevents definitive conclusions about whether observed improvement of living environment preceded plague risk. It is possible that better living conditions contribute to reduced exposure to plague vectors. Alternatively, areas with historically lower plague burden may have experienced greater infrastructural improvements. Nevertheless, our findings are consistent with known mechanisms of vector-borne disease transmission, offering valuable insights for longitudinal study designs that could resolve these questions in future research. In addition, because participant recruitment relied on community mobilization and outreach within each village, individuals who were absent or less socially engaged at the time of the survey may have been underrepresented. Although the surveyed population covered the majority of residents present in each community, this potential selection bias may have led to modest overestimation of awareness or protective behaviors. Such limitations should be considered when interpreting the anthropogenic indices and their associations.

In conclusion, our findings pointed at the improved living environment as the most likely anthropogenic driver that is associated with the reduced risk of plague occurrence. The effective management of plague risk requires a holistic approach that integrates multifaceted natural and anthropogenic factors such as sanitation, protective behavior, and environmental conditions. Crucially, our findings implicate future investigations of community perceptions of zoonotic diseases risk and preventive practices in a broader range of socioeconomic settings.

## Ethics statements

The procedures and protocols for sample collection and processing in this study were reviewed and approved by the Medical Ethics Committee of the Yunnan Institute of Endemic Disease Control and Prevention.

## CRediT authorship contribution statement

**Zhe Lou:** Writing – original draft, Investigation, Formal analysis, Data curation. **Huajun Zhao:** Writing – review & editing, Investigation, Formal analysis. **Chao Su:** Methodology, Investigation, Data curation. **Ennian Pu:** Methodology, Investigation, Data curation. **Xiyang Li:** Writing – original draft, Investigation, Formal analysis, Data curation. **Qingxi Shi:** Investigation. **Yunqin Shen:** Investigation. **Ying Zhao:** Investigation. **Zihou Gao:** Writing – review & editing, Supervision, Funding acquisition, Conceptualization. **Ruiyun Li:** Writing – review & editing, Supervision, Funding acquisition, Conceptualization.

## Declaration of competing interest

They authors declare that they have no competing interests.

## Data Availability

The datasets used and/or analysed during the current study are available from the corresponding author on reasonable request.

## Appendix A Supplementary data

Supplementary material Image 1
