# Supplementary material for: Influence of natural and anthropogenic drivers on plague risk in Southwest China: A multicenter cross-sectional study
Source: One Health. 2025 Jul 25;21:101142. doi: 10.1016/j.onehlt.2025.101142 (PMC12319548; doi:10.1016/j.onehlt.2025.101142)
Supplement: Supplementary file 1 — Supplementary material [file mmc1.docx]

**Supplementary Material**

**Supplementary Results**

Respondents of the Achang ethnic group is of an older age profile as compared to thos of the Yi, Dai, and Han groups. Similarly, the median age of the respondents of the Dai group is younger than those of the Hani and Han groups. By contrast, no significant differences were observed in the age profiles of the respondents from the Miao, Han, Hani, and Yi groups.

**Figures**

**Figure S1. Disparities of animal raising behavior across ethnic groups.**

**Tables**

**Table S1. The Living status, household sanitation and Protective behavior synthetic indices and weights.**

| Primary indicator | Secondary indicator | Weight | Attribute |
| --- | --- | --- | --- |
| Living status | road hardening rate | 0.103 | positive |
|  | house structure | 0.343 | positive |
|  | damage to crops by rodents | 0.287 | negative |
|  | rodent activity in the house | 0.267 | negative |
|  |  |  |  |
| Household sanitation | presence of sealed granaries or storage areas | 0.221 | positive |
|  | grain and food storage habits | 0.093 | positive |
|  | firewood stacking methods | 0.090 | negative |
|  | ownership of livestock | 0.595 | negative |
|  |  |  |  |
| Protective behavior | use of pesticides to prevent flea bites | 0.120 | positive |
|  | use of protective gear during agricultural activities | 0.144 | positive |
|  | use of rodenticides at home | 0.415 | positive |
|  | use of flea control sprays at home | 0.321 | positive |

**Table S2. Significance test results for general demographic information among ethnic groups.** Statistically significant results (*p*<0.05) are marked with *.

| **Variable** | **Estimate** | ***p* value** |
| --- | --- | --- |
| Sex^a^ | 41.578 | <0.001* |
| Age^b^ | 384.487 | <0.001* |
| Animal raising^a^ | 123.890 | <0.001* |

^a^X-squared test was used for the assessment of the difference between sex and population subgroups different animal raising behaviors.

^b^The Kruskal-Wallis test was employed to assess age disparities of the respondents.

**Table S3. Pairwise comparisons of ethnic groups on sex.** Statistically significant results (*p*<0.05) are marked with *.

| **Group** | **Dai** | **Hani** | **Yi** | **Lahu** | **Han** | **Naxi** | **Miao** |
| --- | --- | --- | --- | --- | --- | --- | --- |
| **Hani** | 1 |  |  |  |  |  |  |
| **Yi** | 0.006* | 0.971 |  |  |  |  |  |
| **Lahu** | 0.949 | 1 | 1 |  |  |  |  |
| **Han** | 0.107 | 1 | 1 | 1 |  |  |  |
| **Naxi** | <0.001* | 0.032* | 1 | 0.357 | 0.095 |  |  |
| **Miao** | 1 | 1 | 1 | 1 | 1 | 1 |  |
| **Achang** | 1 | 1 | 0.027* | 1 | 0.415 | <0.001* | 1 |

**Table S4. Pairwise comparisons of ethnic groups on age.** Statistically significant results (*p*<0.05) are marked with *.

|  | **Kruskal-Wallis chi-squared** | **Adjusted *p* value** |
| --- | --- | --- |
| Achang-Miao | 1.297827 | 1 |
| Achang-Hani | 1.891911 | 0.819 |
| Miao-Hani | -0.113598 | 1 |
| Achang-Yi | 3.460297 | 0.008* |
| Miao-Yi | 0.952868 | 1 |
| Hani-Yi | 1.693607 | 1 |
| Achang-Lahu | 7.140485 | <0.001* |
| Miao-Lahu | 3.214103 | 0.02* |
| Hani-Lahu | 5.435322 | <0.001* |
| Yi-Lahu | 3.520745 | 0.006* |
| Achang-Dai | 5.822457 | <0.001* |
| Miao-Dai | 2.179547 | 0.410 |
| Hani-Dai | 3.944502 | 0.001* |
| Yi-Dai | 1.938499 | 0.736 |
| Lahu-Dai | -1.938771 | 0.735 |
| Achang-Han | 2.982054 | 0.040* |
| Miao-Han | 0.315299 | 1 |
| Hani-Han | 0.798395 | 1 |
| Yi-Han | -1.231382 | 1 |
| Lahu-Han | -5.681841 | <0.001* |
| Dai-Han | -3.983756 | <0.001* |
| Achang-Naxi | 15.162418 | <0.001* |
| Miao-Naxi | 7.686657 | <0.001* |
| Hani-Naxi | 13.65709 | <0.001* |
| Yi-Naxi | 11.121365 | <0.001* |
| Lahu-Naxi | 7.635929 | <0.001* |
| Dai-Naxi | 10.635818 | <0.001* |
| Han-Naxi | 16.230992 | <0.001* |

**Table S5. Pairwise comparisons of ethnic groups on animal raising.** Statistically significant results (*p*<0.05) are marked with *.

| **Group** | **Dai** | **Hani** | **Yi** | **Lahu** | **Han** | **Naxi** | **Miao** |
| --- | --- | --- | --- | --- | --- | --- | --- |
| **Hani** | <0.001* |  |  |  |  |  |  |
| **Yi** | 0.886 | 0.002* |  |  |  |  |  |
| **Lahu** | 0.071 | 0.014* | 1 |  |  |  |  |
| **Han** | 0.245 | <0.001* | 1 | 1 |  |  |  |
| **Naxi** | <0.001* | 1 | <0.001* | <0.001* | <0.001* |  |  |
| **Miao** | 1 | 1 | 1 | 1 | 1 | 0.051 |  |
| **Achang** | 1 | <0.001* | 1 | 1 | 1 | <0.001* | 1 |

**Table S6. Exposure to plague associated risk factors among respondents.**

| **Exposure type** | **Exposed (n, %)** | **Not Exposed (n, %)** |
| --- | --- | --- |
| Self-dead rodents | 319 (10.64%) | 2660 (88.73%) |
| Flea bites | 460 (15.34%) | 2523 (84.16%) |
| Contact with plague patient | 18 (0.60%) | 2928 (97.67%) |

**Table S7. Significance test results for living status, household sanitation, and protective behavior among ethnic groups.** Statistically significant results (*p*<0.05) are marked with *.

| **Variable** | **Estimate** | ***p* value** |
| --- | --- | --- |
| Living status^a^ | 0.969 | 0.465 |
| Household sanitation^b^ | 13.707 | 0.057 |
| Protective behavior^a^ | 2.663 | 0.021* |

^a^ANOVA was used for the assessment of the difference between sex and protective behavior.

^b^The Kruskal-Wallis test was employed to assess household sanitation disparities of the respondents.

**Table S8. Pairwise comparisons of ethnic groups on protective behavior.** Statistically significant results (*p*<0.05) are marked with *.

| **Group** | **Dai** | **Hani** | **Yi** | **Lahu** | **Han** | **Naxi** | **Miao** |
| --- | --- | --- | --- | --- | --- | --- | --- |
| **Hani** | 1 |  |  |  |  |  |  |
| **Yi** | 1 | 1 |  |  |  |  |  |
| **Lahu** | 0.485 | 0.877 | 0.146 |  |  |  |  |
| **Han** | 1 | 1 | 1 | 0.047* |  |  |  |
| **Naxi** | 1 | 1 | 0.697 | 1 | 0.468 |  |  |
| **Miao** | 1 | 1 | 1 | 0.697 | 1 | 1 |  |
| **Achang** | 1 | 1 | 1 | 1 | 1 | 1 | 1 |

**Plague Natural Epidemic Foci High-Risk Population Survey Form**

**Survey Number:**

**1. General Information**

1.1 Name:

1.2 ID Number:

1.3 Gender: (1) Male (2) Female

1.4 Age:

1.5 Occupation:(1) Nursery Child (2) Non-household Children (3) Student (4) Medical and Health Personnel (5) Teacher (6) Caretaker/Nanny (7) Catering Industry (8) Commercial Services (9) Worker (10) Migrant Worker (11) Farmer (12) Shepherd (13) Fisherman/Boatman (14) Cadre/Staff (15) Retired/Resigned (16) Homemaker/Unemployed (17) Other

1.6 Current Residence (complete details):

Province

City

County/District

Town/Street

Village

1.6.1 Phone Number:

1.7 Work Unit:

**2. Plague Exposure History**

2.1 Has there been a plague outbreak in your place of residence (village)?

(1) Yes

(2) No

2.2 Have you ever come into contact with a self-dead rodent?

(1) Yes

(2) No

2.3 Have you ever been bitten by fleas?

(1) Yes

(2) No

2.4 Have you contacted with a plague patient?

(1) Yes

(2) No

**3. Living Environment Survey**

3.1 Are the roads surrounding your house surfaced with concrete?

(1) Yes

(2) No

3.2 Type of House Structure:

(1) Simple Earth/Wooden House

(2) Brick-Wood House

(3) Brick-Concrete House

(4) Independent Yard or Villa

(5) Other

3.3 Do you have a closed granary or storage area for grains?

(1) Yes

(2) No

3.4 Grain and Food Storage Habits:

(1) Stored casually

(2) Stored centrally

(3) Hung

(4) Other

3.5 How is firewood stored in your home?

(1) Stored casually

(2) Stored neatly

(3) No firewood

3.6 Months when rodent activity near your house or farmland significantly increased: ____

3.7 Have you noticed signs of rodent activity near your home (e.g., damage to crops)?

(1) Yes

(2) No

3.8 Have you noticed signs of rodent activity inside your home (e.g., stealing food, damaging furniture)?

(1) Yes

(2) No

3.9 Do you raise pets, poultry, or livestock?

(1) Yes

(2) No

Types (Quantity): Chicken (), Duck (), Pig (), Sheep (), Cow (), Goose (), Dog (), Rabbit (), Horse (), Cat ()

3.9.1 Do the pets, poultry, and livestock have separate enclosures?

(1) Yes

(2) No

3.9.2 Are the enclosures located inside or outside your house?

(1) Inside

(2) Outside

3.10 Have you found fleas on any animals in your home?

(1) Yes

(2) No

3.11 Main crops planted in your home:

Corn, Potato, Rice, Wheat, Cabbage, Kidney Beans, Oilseed

3.12 Main crops planted near your house:

Corn, Potato, Rice, Wheat, Cabbage, Kidney Beans, Oilseed

**4. Plague Prevention Awareness**

4.1 Do you know plague?

(1) Yes

(2) No

4.2 Have you ever used insecticides or repellents to prevent flea bites?

(1) Yes

(2) No

4.3 Do you wear protective gear, like rain boots, when doing agricultural activities?

(1) Always

(2) Occasionally

(3) No

4.4 Have you ever used rodenticides at home?

(1) Yes

(2) No

4.4.1 How often do you use rodenticides?

(1) Once a year

(2) Twice a year

(3) Three times or more a year

(4) No fixed schedule, used when rodents are abundant

4.5 Have you ever sprayed flea and insecticides at home?

(1) Yes

(2) No

4.5.1 How often do you use flea and insecticides?

(1) Once a year

(2) Twice a year

(3) Three times or more a year

(4) No fixed schedule, used when rodents are abundant

**5. Health Status and Medical Treatment**

5.1 Have you recently had symptoms like fever, swollen lymph nodes, or cough?

(1) Yes

(2) No

5.2 Have you ever sought medical treatment or been hospitalized due to symptoms like fever, swollen lymph nodes, or cough?

(1) Yes

(2) No

5.3 Have you ever been diagnosed with plague or suspected of having plague?

(1) Yes

(2) No

**Survey Unit:**

**Survey Date:**

**Surveyor’s Signature:**

**Survey Form Instructions:**

1. Please use a black ink pen, and write neatly.

2. All numbers should be written in Arabic numerals (e.g., 0, 1, 2, 3, …).

3. For all dates, fill in to the day (e.g., April 5, 2023, should be entered as 20230405).
